# Neuronal‐specific proteasome augmentation via Prosβ5 overexpression extends lifespan and reduces age‐related cognitive decline

**DOI:** 10.1111/acel.13005

**Published:** 2019-07-23

**Authors:** Erin Munkácsy, E. Sandra Chocron, Laura Quintanilla, Christi M. Gendron, Scott D. Pletcher, Andrew M. Pickering

**Affiliations:** ^1^ The Sam and Ann Barshop institute for Longevity and Aging Studies UT Health San Antonio San Antonio Texas; ^2^ Department of Molecular Medicine UT Health San Antonio San Antonio Texas; ^3^ Department of Molecular and Integrative Physiology and the Geriatrics Center University of Michigan Ann Arbor Michigan; ^4^ The Glenn Biggs Institute for Alzheimer's & Neurodegenerative Diseases UT Health San Antonio San Antonio Texas

**Keywords:** aging, *Drosophila*, neurodegeneration, proteasome

## Abstract

Cognitive function declines with age throughout the animal kingdom, and increasing evidence shows that disruption of the proteasome system contributes to this deterioration. The proteasome has important roles in multiple aspects of the nervous system, including synapse function and plasticity, as well as preventing cell death and senescence. Previous studies have shown neuronal proteasome depletion and inhibition can result in neurodegeneration and cognitive deficits, but it is unclear if this pathway is a driver of neurodegeneration and cognitive decline in aging. We report that overexpression of the proteasome β5 subunit enhances proteasome assembly and function. Significantly, we go on to show that neuronal‐specific proteasome augmentation slows age‐related declines in measures of learning, memory, and circadian rhythmicity. Surprisingly, neuronal‐specific augmentation of proteasome function also produces a robust increase of lifespan in *Drosophila melanogaster*. Our findings appear specific to the nervous system; ubiquitous proteasome overexpression increases oxidative stress resistance but does not impact lifespan and is detrimental to some healthspan measures. These findings demonstrate a key role of the proteasome system in brain aging.

## 1 INTRODUCTION, RESULTS, AND DISCUSSION

**Figure 1:**
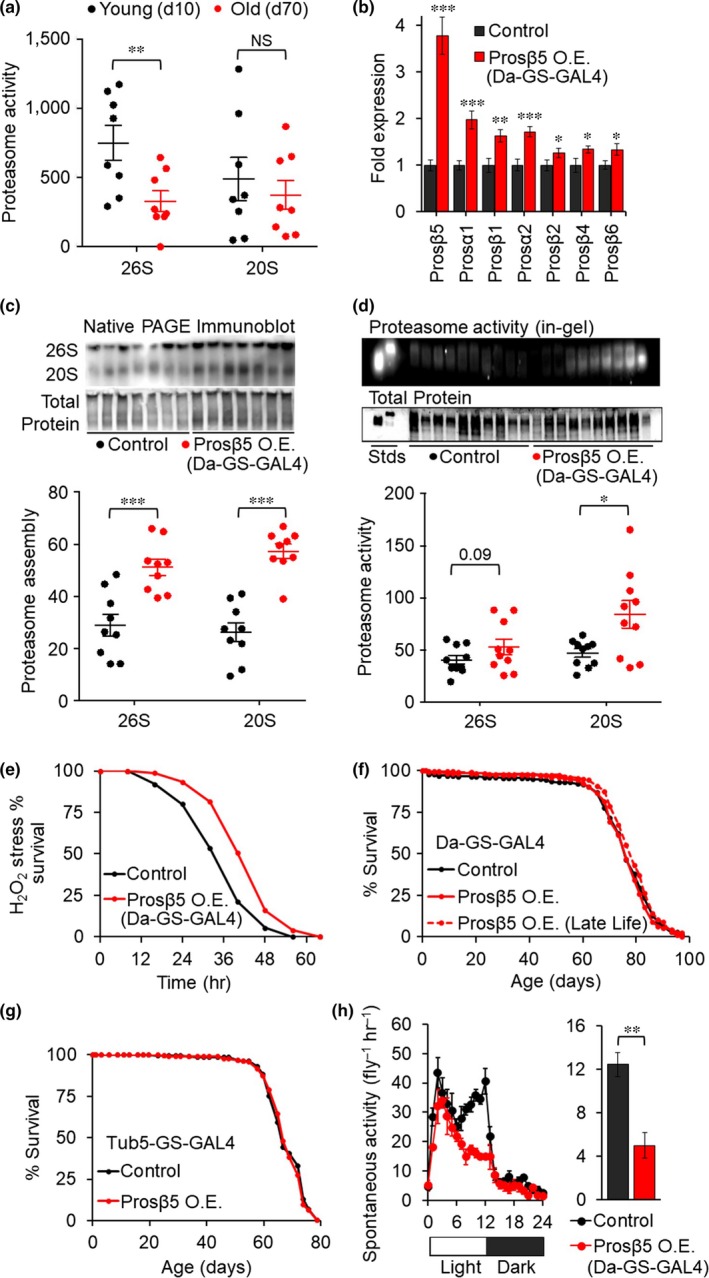
Prosβ5 drives upregulation of multiple proteasome subunits and increases proteasome function but does not extend lifespan when ubiquitously overexpressed. (a) Proteasome activity declines with age in W^1118^ fly heads, *N* = 8. (b) Overexpression of Prosβ5 through Da‐GS‐GAL4>UAS‐Prosβ5 ± 200 μM RU486 increases mRNA expression of multiple 20S core proteasome subunits in day 10 female flies, *N* = 8. (c) Prosβ5 overexpression increases assembly of 20S and 26S proteasome, Native PAGE immunoblot, values normalized to total protein, based on India ink stain, *N* = 9. (d) Prosβ5 overexpression increases 20S proteasome based on an in‐gel Suc‐LLVY‐AMC activity overlay assay, values normalized to total protein, based on Coomassie stain, *N* = 10. (e) Prosβ5 overexpression under control of the driver Da‐GS‐GAL4 increases oxidative stress resistance. Flies were fed 4.4 M H_2_O_2_ mixed with 5% sucrose, and survival was monitored every 8 hr, *N* = 75. Flies were removed from RU486 during stress assay to prevent potential differences in consumption. (f, g) *Drosophila* lifespan is not increased by Prosβ5 overexpression under either of the ubiquitous drivers Da‐GS‐GAL4 or Tub5‐GS‐GAL4 ± 200 μM RU486, *N* = 200. (h) Prosβ5 overexpression under control of the driver Da‐GS‐GAL4 reduces healthspan in flies based on spontaneous activity measures at day 50, *N* = 3–4 vials with 25 flies per vial. Logrank evaluations for lifespans are included in Figure S1 Whole uncropped immunoblot images are provided in Figure S2. NS *p*>0.05, *p<0.05, **  *p*<0.01, ***p<0.001. Significance is based on Students T‐test. Values are Mean ± SEM.

With age, there is a progressive decline in 26S proteasome function in the nervous system of mammals (Keller, Hanni, & Markesbery, 2000) as well as flies (Figure 1a), with a corresponding increase in 20S proteasome levels but not activity, which either declines or is unchanged (Figure 1a; Keller et al., 2000; Tonoki et al., 2009; Vernace, Arnaud, Schmidt‐Glenewinkel, & Figueiredo‐Pereira, 2007). These changes likely result from reduced capacity of the existing proteasome (Bulteau, Petropoulos, & Friguet, 2000), diminished 26S assembly (Tonoki et al., 2009; Vernace et al., 2007) and disassembly of the 26S proteasome into free 20S to compensate for reduced 20S functionality. It has been shown that proteasome depletion and inhibition in mice can mirror brain aging phenotypes, producing neurodegeneration, cognitive deficits, and formation of Lewy‐like bodies (Bedford et al., 2008; Romero‐Granados, Fontan‐Lozano, Aguilar‐Montilla, & Carrion, 2011). The goal of this study is to establish whether age‐related cognitive decline can be ameliorated by augmenting proteasome function.

The size and complexity of the proteasome has made manipulating its expression a challenge. Elevating the proteasome β5 subunit increases both expression of other subunits and whole proteasome assembly in mammalian cell cultures (Chondrogianni et al., 2005; Liu et al., 2007) and *Caenorhabditis elegans* (Chondrogianni, Georgila, Kourtis, Tavernarakis, & Gonos, 2015). We used the same approach in *Drosophila melanogaster*, utilizing UAS‐Prosβ5 (fly ortholog of the β5 subunit; Staudt et al., 2005). We used the mifepristone (RU486) inducible GeneSwitchGAL4 driver system to limit gene overexpression to adulthood, thereby removing developmental artifacts and allowing experiment and control animals to be genetically identical siblings. Overexpression of Prosβ5 posteclosion increased mRNA of other core proteasome subunits (Figure 1b), enhanced proteasome assembly (Figure 1c and Figure S1) and activity (Figure 1d), and increased oxidative stress resistance (Figure 1e), independent of artifacts from RU486 treatment (Figure S2). Despite improvements to oxidative stress resistance, ubiquitous elevation of proteasome function did not impact lifespan when induced either throughout adulthood or in late life (from 40 days posteclosion) (Figure 1f,g; Figures S3 and S4). This finding was confirmed using two independent ubiquitous driver lines (Da‐GS‐GAL4 and Tub5‐GS‐GAL4). In addition, we did not observe any improvements in healthspan and instead found reduced spontaneous activity in middle‐aged flies providing some indication of toxicity (Figure 1h). Our findings conflict with a recent report that Prosβ5 under control of Da‐GS‐GAL4 extends lifespan (Nguyen et al., 2019). We note that a considerably lower dose of RU486 was employed in the study by Nguyen and colleagues (~23 μM in contrast to 200 μM in the present study). Our different results may stem from differences in levels of transgene induction; it is possible that low‐level ubiquitous proteasome overexpression is beneficial while higher levels may be detrimental.

**Figure 2:**
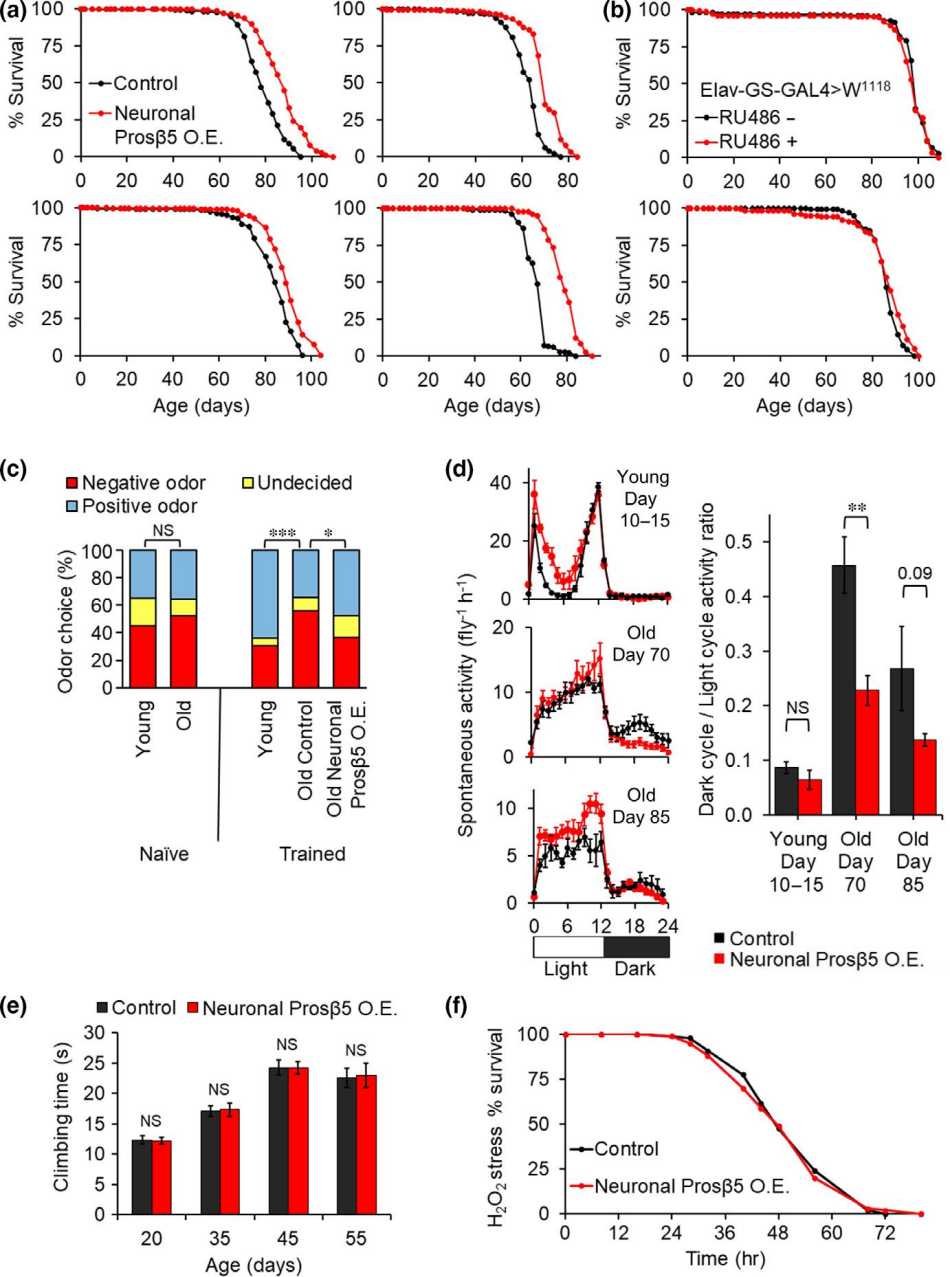
Neuronal‐specific Prosβ5 overexpression extends lifespan and reduces age‐related deficits in learning, memory, and brain function. (a) Neuronal Prosβ5 overexpression (Elav‐GS‐GAL4>UAS‐Prosβ5 ± 200 μM RU486) extends female fly lifespan. Evaluations are based on four independent lifespan assays, *N* = 200–250 each. (b) RU486 alone does not extend lifespan in the genetic background evaluated. Lifespan measure of Elav‐GS‐GAL4>W^1118^ flies ± 200 μM RU486. UAS‐Prosβ5 flies were backcrossed into the evaluated W^1118^ strain prior to the start of this investigation. (c) Neuronal Prosβ5 overexpression reduces age‐related cognitive deficits in olfaction aversion training. Experiments performed as in Malik and Hodge (2014) *N* = 150. (d) Age disrupts circadian rhythmicity. Neuronal Prosβ5 overexpression reduces declines in circadian rhythmicity, *N* = 125. (e) Neuronal Prosβ5 overexpression does not improve muscle function evaluated through climbing capacity. *N* = 100. (f) No improvement in oxidative stress resistance observed with neuronal‐specific Prosβ5 overexpression. Logrank evaluations for lifespans are included in Figure S1. NS *p*>0.05, *p<0.05, **p<0.01, ***p<0.001. Significance is based on Students T‐test except panel C where significance is based on Chi‐Sq test. Values are Mean ± SEM

In contrast, when Prosβ5 overexpression was limited to the nervous system (using the pan‐neuronal driver Elav‐GS‐GAL4), we observed a robust extension in median and maximum lifespan which was reproduced across four independent cohorts (Figure 2a and Figure S5). Because of concerns regarding potential off‐target effects from RU486 on *Drosophila* lifespan (Landis et al., 2015), we undertook parallel experiments in flies of the same genetic background, minus the UAS‐Prosβ5 transgene, and found no impact of RU486 treatment on lifespan (Figure 2b and Figure S5). Thus, we can conclude that the extension in lifespan from pan‐neuronal Prosβ5 overexpression is not an artifact of RU486 treatment. Importantly, pan‐neuronal Pr

osβ5 overexpression not only extended lifespan but also reduced age‐related cognitive deficits. We demonstrated improvements in learning and memory in aged animals using olfaction aversion training. Animals were exposed in alternation to two neutral odors (3‐octanol & 4‐methylcyclohexanol), one of which was paired with exposure to a mild electric shock. After five training rounds, animals were permitted to recover for one hour and then placed in a T‐maze with opposing odors from either side (Malik & Hodge, 2014). While young (10 days posteclosion) animals showed a significant increase in avoidance of the “negative” odor after training, old (70 days posteclosion) animals showed no increase in avoidance after training. However, *Drosophila* with neuronal Prosβ5 overexpression continued to show a posttraining increase in avoidance of the “negative” odor at old age (Figure 2c), demonstrating a retention of associative learning ability. In both humans and animal models, circadian rhythmicity is well‐established as correlating with and potentially contributing to age‐related cognitive decline (Antoniadis, Ko, Ralph, & McDonald, 2000). *Drosophila* show a defined activity distribution with high activity during the day and low activity at night. With age, this pattern becomes less defined. We found this rhythmicity deficit to be partially prevented in flies which overexpressed Prosβ5 (Figure 2d). Significantly, age‐related declines in climbing capacity were not altered in these animals. This suggests that the improvements in activity measures are independent of muscle function (Figure 2e). Additionally, the animals showed no increase in oxidative stress resistance when fed hydrogen peroxide, further supporting a neuronal‐specific role rather than whole body adaptation (Figure 2f). Furthermore improvements appear independent of impact from RU486‐induced off‐target effects. No increase in proteasome activity or behavioral changes were observed under treatment with RU486 in the absence of the psmb5 transgene (Figure S6).

Our findings demonstrate that pan‐neuronal augmentation of proteasome function can ameliorate age‐related cognitive decline, specifically in learning, memory, and circadian rhythmicity. We also show that pan‐neuronal proteasome overexpression reproducibly extends lifespan while ubiquitous proteasome overexpression did not improve lifespan and may be detrimental to healthspan. This finding underscores the importance of the nervous system and neuronal proteasome function as determinants of lifespan in *Drosophila*.

## 2 EXPERIMENTAL PROCEDURES

Details provided in supplemental files.

## CONFLICT OF INTEREST

None declared.
