# Supplementary material for: Neuronal‐specific proteasome augmentation via Prosβ5 overexpression extends lifespan and reduces age‐related cognitive decline
Source: Aging Cell. 2019 Jul 23;18(5):e13005. doi: 10.1111/acel.13005 (PMC6718538; doi:10.1111/acel.13005)
Supplement: Supplementary file 1 [file ACEL-18-e13005-s001.pdf]

Native PAGE immunoblot (uncropped)
anti-proteasome

Protein loading
(india ink stain)

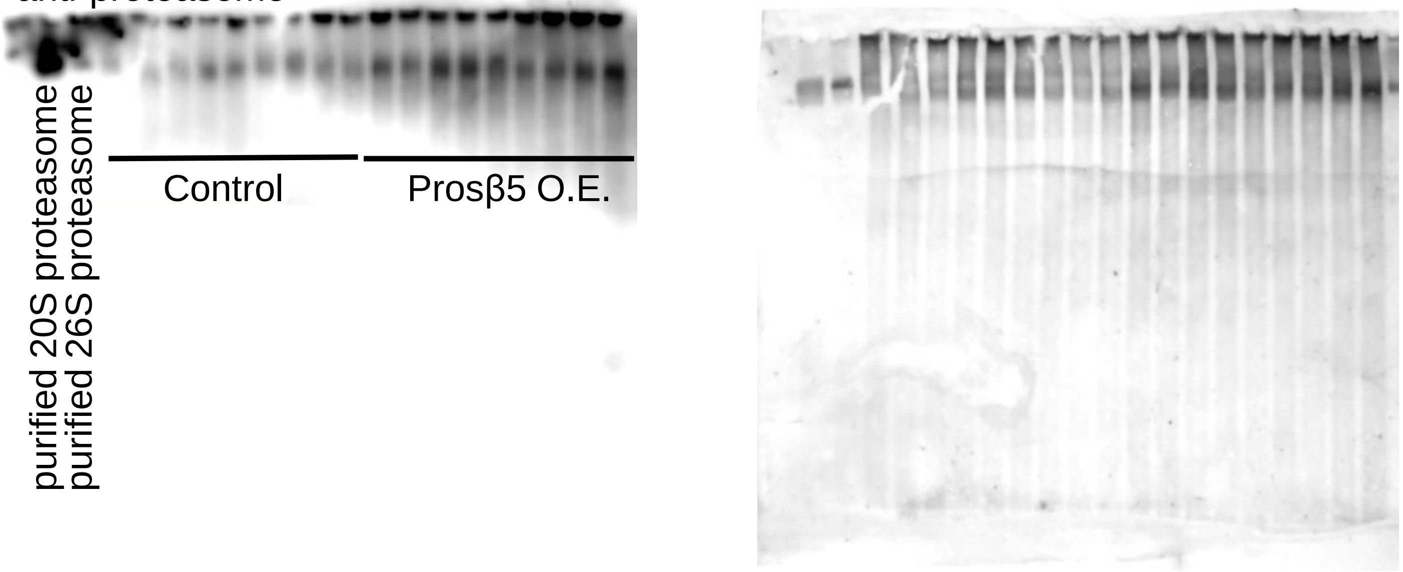

Supplemental Figure 1:
Uncropped Native PAGE immunoblot from Figure 1C

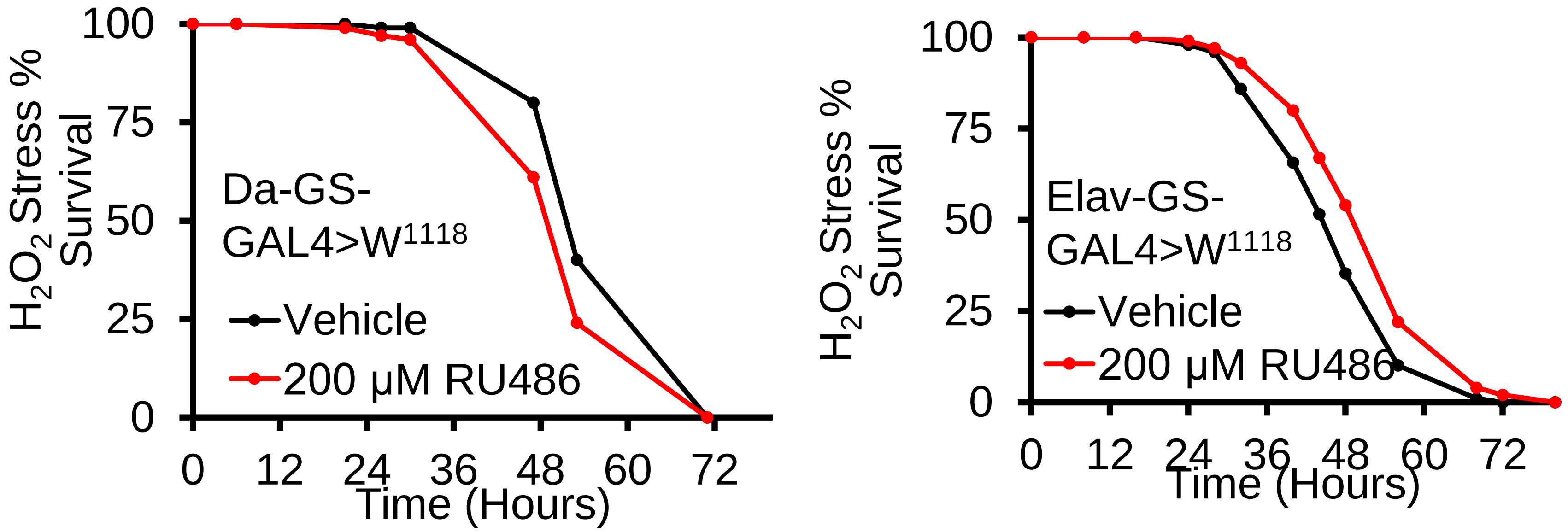

Supplemental Figure 2:
H_2O_2 stress survival assay RU486 controls for Figures 1E and 2F. RU486 treatment produces minimal changes to oxidative stress under control of the drivers Da-GS-GAL4 and Elav>GS-GAL4. Flies were fed 4.4M H_2O_2 mixed with 5% sucrose, survival was monitored every 8 hr, N = 75. Flies were removed from RU486 during stress assay to prevent potential differences in consumption.

	- RU486	+ RU486	%	Chi-Square	Logrank
DA-GS-GAL4>UAS-Prosβ5<br>N ≈ 200<br>Median (50%)<br>Maximum (10%)	 <br>75.4<br>87.1	 <br>75.3<br>84.9	 <br>-0.2<br>-2.6	 <br>2.5	 <br>0.11
Tub5-GS-GAL4>UAS-Prosβ5<br>N ≈ 250<br>Median (50%)<br>Maximum (10%)	 <br>66.0<br>74.7	 <br>66.5<br>73.7	 <br>0.8<br>-1.3	 <br>0.2	 <br>0.64

Supplemental Figure 3:
Lifespan statistics for Figures 1F-G.

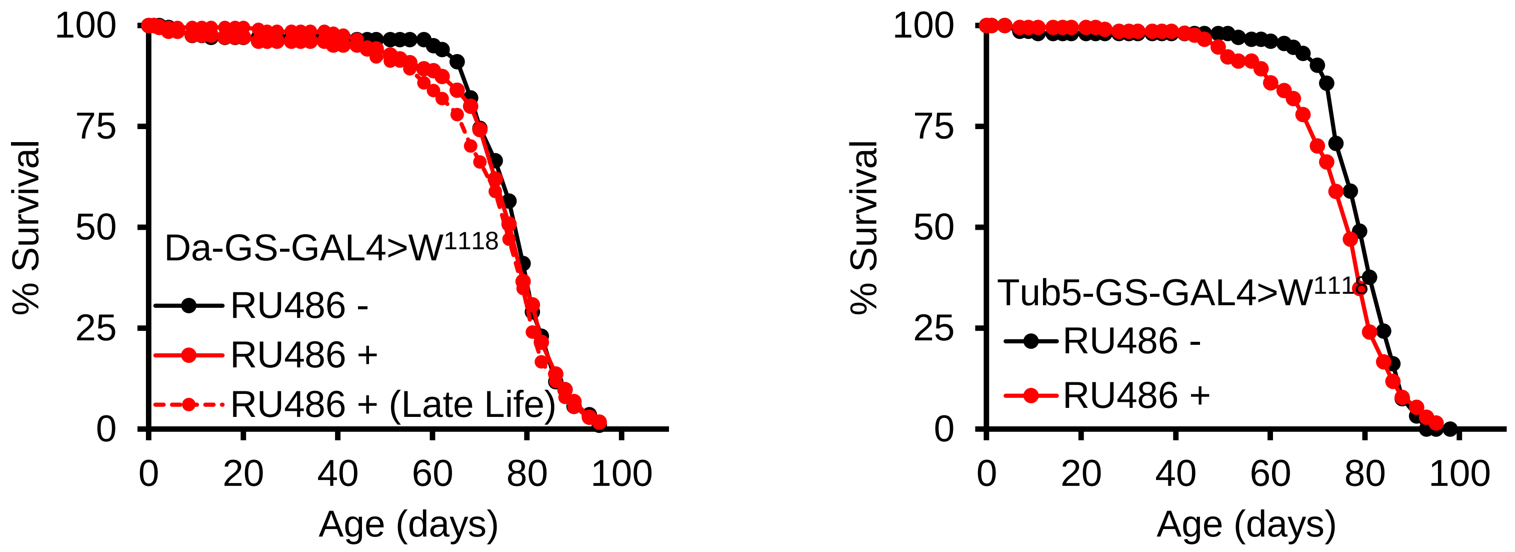

Supplemental Figure 4:
Lifespan RU486 controls for Figure 1F-G.

	Elav-GS-GAL4>UAS-Prosβ5				
	- RU486	+ RU486	%	Chi-Square	Logrank
Lifespan 1 N ≈ 250					
Median (50%)	77.4	86.5	11.9	66	4.44×10^{-16}
Maximum (10%)	89.1	98.4	10.4		
Lifespan 2 N ≈ 175					
Median (50%)	63.3	68.7	8.5	77.8	$<1 \times 10^{-18}$
Maximum (10%)	68.7	77.6	12.9		
Lifespan 3 N ≈ 225					
Median (50%)	67.0	77.8	16.1	176	$<1 \times 10^{-18}$
Maximum (10%)	70.0	85.3	21.7		
Lifespan 4 N ≈ 250					
Median (50%)	83.9	88.9	5.9	22.6	2.03×10^{-6}
Maximum (10%)	93.1	99.2	6.5		

	Elav-GS-GAL4>W^{1118}				
	- RU486	+ RU486	%	Chi-Square	Logrank
Lifespan 1 N ≈ 175					
Median (50%)	98.0	97.3	-0.7	2.4	0.12
Maximum (10%)	104.5	104.4	-0.2		
Lifespan 2 N ≈ 175					
Median (50%)	85.5	86.6	1.2	77.8	0.04
Maximum (10%)	92.2	95.6	3.6		

Supplemental Figure 5:
Lifespan statistics for Figures 2A-B.

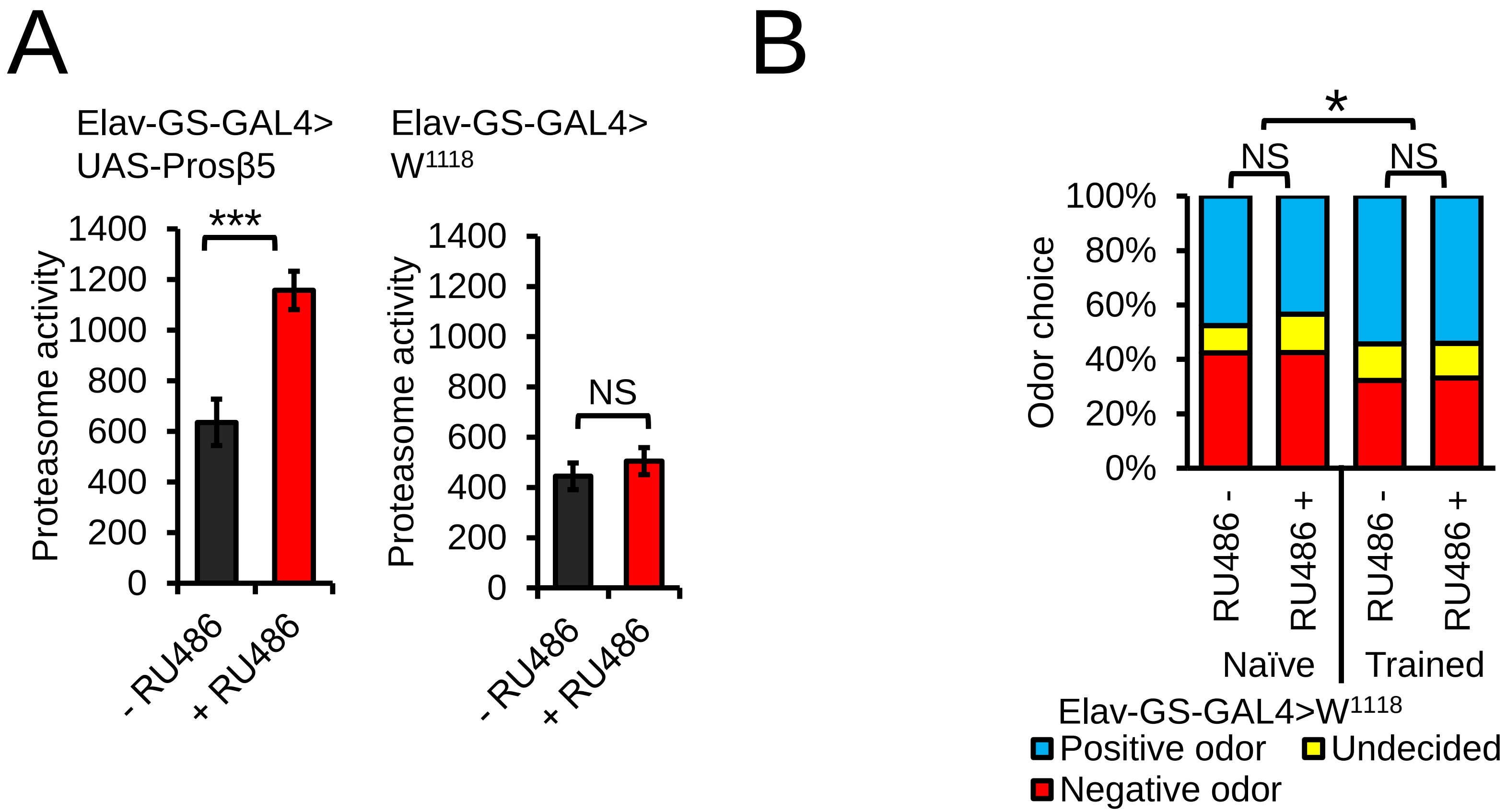

Supplemental Figure 6:

A. RU486-induced Prosβ5 overexpression increases under control of the Elav-GS-GAL4 (Elav-GS-GAL4>Prosβ5) driver increases 20S proteasome based on an Suc-LLVY-AMC activity assay. No increase in activity seen in flies lacking the transgene (Elav-GS-GAL4>W^{1118}), N = 12. **B**. RU486 treatment does not alter behavior in olfaction aversion training. Assay performed in 10 day old Elav-GS-GAL4>W^{1118} flies, N = 150.
