# Supplementary material for: Neuronal‐specific proteasome augmentation via Prosβ5 overexpression extends lifespan and reduces age‐related cognitive decline
Source: Aging Cell. 2019 Jul 23;18(5):e13005. doi: 10.1111/acel.13005 (PMC6718538; doi:10.1111/acel.13005)
Supplement: Supplementary file 2 [file ACEL-18-e13005-s002.pdf]

Supplemental Experimental Procedures

Fly Lines and Strain Maintenance

UAS-ProsBeta5 (Staudt, Molitor et al. 2005) stocks were obtained from the Bloomington Drosophila Stock Center (NIH P40OD018537). All lines were backcrossed into our lab W^{1118} background strain for eight generations.

All lines were maintained on agar-cornmeal-dextrose-yeast growth media (Ren, Finkel et al. 2009) in a humidified 24°C incubator with 12:12-hour light:dark cycle. All crosses were set up with female virgins of the respective GAL4 driver line and male UAS-ProsBeta5 or W^{1118} flies. Progeny were collected within 48 hours of eclosion and allowed to mate on 10% sugar/yeast (SY10) media (Skorupa, Dervisefendic et al. 2008) for another 48 hours. After this period, females were separated and sorted into sets of 25 flies per vial containing SY10 media supplemented either with 200 µM mifepristone (RU486) or ethanol vehicle, mixed directly into the food. 8 µM Blue Dye #1 was added to food containing RU486 for the purpose of identification. Carbon dioxide was used to briefly anesthetize flies for sorting. Flies were moved to vials of fresh media every two to three days.

Quantitative PCR

mRNA was isolated from individual flies using standard Trizol method and cDNA prepared using High-capacity cDNA reverse transcriptase kit (Applied Biosystems). Quantitative PCR was carried out using SYBR Green and normalized to beta-actin using the following primer pairs: Actin79β (CATCCGCAAGGATCTGTATG, TTCCTTTTGCATACGGTCAG), Prosα1 (CCAGGTGTACACGCAGAATG, TAGCTGTGGGCCTCCAGAGT) Prosα2 (TCTACCAATCCGATCCTTCG, GGCAGTCATTTTTCCCTCAA), Prosβ1 (GATCAATATGCAGCCCG-ACT, AGCCAGTAGGCCACAATGTC), Prosβ2 (GTAATGGATCTTGGCGCAGT, GCAATGCAACTCTTTTGAA) Prosβ4 (ATGGCTATGGTGCGATCTTC, GCTGATTGGCTCCAAATCTC), Prosβ5 (ATTGCGTTTACTGGGAC-AGG, GATCCCTCGGAGTCCACATA), Prosβ6 (GCAGAGCTACGAGCATACCC, GTAGCCTTCTCGCAGTGACC).

Plate-based Proteasome Activity Assay

Flies were individually homogenized by pestle in 100 µL chilled proteasome buffer (50 mM Tris, 5 mM $MgCl_2$, 1 mM DTT, pH 7.4). Samples were vortexed and then centrifuged at 21,000g at 4°C for 15 minutes. In a black 96-well plate, 10 µL of supernatant was added to 80 µL proteasome buffer supplemented with either an additional 5 mM ATP for measuring 26S proteasome activity or 0.05% SDS for measuring 20S activity. Finally, 50 µM Suc-LLVY-AMC fluorogenic substrate in 10 µL proteasome buffer was added to each well to measure chymotrypsin-like activity. The plate was incubated at 37°C in a SpectrumMax M2 plate reader for four hours with fluorescence measured every 10 minutes with 355 nm excitation and reading 460 nm emission. Total proteasome activity per sample was defined as the delta between initial and final measurements.

Proteasome In-gel Activity and Immunoblot

Flies were individually homogenized by pestle in 100 µL chilled proteasome buffer (50 mM Tris, 5 mM $MgCl_2$, 1 mM DTT, pH 7.4). Samples were vortexed and then centrifuged at 21,000g at 4°C for 15 minutes. Supernatant was run on 10% Tris-Glycine non-denaturing polyacrylamide gels (Biorad) in Native Gel Buffer (Diluted from 20X; Life Technologies) supplemented with 5 mM $MgCl_2$, 1 mM DTT, and 2 mM ATP to maintain proteasome assembly. Gels were run on ice in a 4ºC cold room at 100V for 2 hours followed by an additional 3 hours at 250V.

To assay proteasome assembly, gels were soaked in running buffer supplemented with 5% SDS for 30 minutes then transferred to PVDF membrane and probed with anti-proteasome α (SCBT, sc-65755). Membranes were visualized with enhanced chemiluminescence (Pierce ECL Western Blotting Substrate) using an Amersham Imager 680 (GE, Healthcare) and then quantified with ImageJ. India ink stain was used as a total protein loading control. ±*

To assay in-gel proteasome chymotrypsin-like activity, gels were incubated in 10mM Suc-LLVY-AMC substrate dissolved in 50mM Tris pH 8.0, 5 mM $MgCl_2$, 1mM DTT, 2 mM ATP for 30 minutes at 37°C. Fluorescence was measured with an ImageQuant 4000 (GE Healthcare), using 312 nm excitation and measuring emissions between 585 and 625 nm (the tail end of the Suc-LLVY-AMC emission spectrum). Purified 20S and 26S proteasomes (Enzo Life Sciences) were used as positive controls. Coomassie stain was used as a total protein loading control.

Hydrogen Peroxide Survival
After 13 days on food supplemented with either 200 µM mifepristone (RU486) or ethanol vehicle (15 days post eclosion), flies were transferred to vials containing half a Kimwipe soaked in 1 mL of 5% sucrose and 4.4M H_2O_2 (no additional. The flies continued to be maintained in a humidified 24°C incubator with 12:12-hour light:dark cycles as described above. Survival was scored every eight hours until all flies were dead and evaluated by logrank analysis.

Lifespan
Lifespan cohorts were set-up as described above, with a minimum of seven vials of 25 flies each. Flies were transferred to fresh media and survival scored every two to three days. dLife software (Linford, Bilgir et al. 2013) was used to record survival and to compare median and maximum lifespan via Logrank analysis. Vials were randomized in terms of tray position and semi-blinded to reduce impacts of environment or investigator bias.

Olfactory Aversion Training
Experiments were performed broadly as described in (Malik and Hodge 2014). Animals were exposed (via an air pump) in alternation to two neutral odors (3-octanol and 4-methylcyclohexanol, prepared as a 1/10 dilution in mineral oil) for 5 minutes under low red-light and a 100V 60Hz shock was applied during exposure to one of the two odors. The odor associated with the electric shock was alternated between vials. After three training rounds per odor, animals were given one hour to recover then placed in a T-maze (Celexplorer labs) with opposing odors from either side. Flies were allowed two minutes to explore the maze after which the maze sections were sealed and the number of flies in each chamber scored. Results were evaluated by chi-square analysis.

Spontaneous Activity and Circadian Rhythm
Spontaneous activity was monitored using a Trikinetic activity monitor, in which vials containing 20-25 flies were secured and activity recorded in a humidified 24°C incubator with 12:12-hour light:dark cycles as described above. Flies were allowed to acclimate for 8 hours prior to data collection. Activity was averaged for each twelve-hour cycle and normalized per fly. Comparisons were done by two-tailed t-test.

Climbing Ability
Vials of 25 flies were transferred to a glass cylinder and allowed to acclimate for 10 minutes. The cylinder was gently tapped to move all flies to the bottom of the chamber. The period of time until 40% (10/25) of the flies had crossed an arbitrary mark placed at 12.5 cm was then recorded. Each vial was evaluated five times and values were averaged between trials. The data presented shows activity up to 55 days post eclosion. Assays were continued until day 65, however, insufficient flies were able to successfully pass the scoring mark at this age.

References

Linford, N. J., C. Bilgir, J. Ro and S. D. Pletcher (2013). "Measurement of lifespan in Drosophila melanogaster." J Vis Exp(71).

Malik, B. R. and J. J. Hodge (2014). "Drosophila adult olfactory shock learning." J Vis Exp(90): e50107.

Ren, C., S. E. Finkel and J. Tower (2009). "Conditional inhibition of autophagy genes in adult Drosophila impairs immunity without compromising longevity." Exp Gerontol **44**(3): 228-235.

Skorupa, D. A., A. Dervisefendic, J. Zwiener and S. D. Pletcher (2008). "Dietary composition specifies consumption, obesity, and lifespan in Drosophila melanogaster." Aging Cell **7**(4): 478-490.

Staudt, N., A. Molitor, K. Somogyi, J. Mata, S. Curado, K. Eulenberg, M. Meise, T. Siegmund, T. Hader, A. Hilfiker, G. Bronner, A. Ephrussi, P. Rorth, S. M. Cohen, S. Fellert, H. R. Chung, O. Piepenburg, U. Schafer, H. Jackle and G. Vorbruggen (2005). "Gain-of-function screen for genes that affect Drosophila muscle pattern formation." PLoS Genet **1**(4): e55.
